# Mass Shootings in the US During the COVID-19 Pandemic

**DOI:** 10.1001/jamanetworkopen.2021.25388

**Published:** 2021-09-16

**Authors:** Pablo A. Peña, Anupam Jena

**Affiliations:** 1 Kenneth C. Griffin Department of Economics, University of Chicago, Chicago, Illinois; 2 Department of Health Care Policy, Harvard Medical School, Boston, Massachusetts; 3 Department of Medicine, Massachusetts General Hospital, Boston; 4 National Bureau of Economic Research, Cambridge, Massachusetts

## Abstract

This cross-sectional study analyzes changes in mass shootings in the US during the COVID-19 pandemic.

## Introduction

Mass shootings are rare events with causes that are not well understood, particularly, the extent to which they are responses to social and economic circumstances. The COVID-19 pandemic imposed sudden and additional psychological and financial strains across society through fear of death, social isolation, economic hardship, and general uncertainty. Although several studies have examined how gun violence, homicides, and other crimes have responded to the pandemic, these analyses have focused on small groups of cities and have not considered mass shootings, which are an extreme form of violence whose causes may not be the same.^1,2,3,4^ This cross-sectional study analyzes changes in mass shootings in the US during the COVID-19 pandemic.

## Methods

This cross-sectional study used publicly available, aggregated data and was exempt from informed consent and human subjects review at Harvard Medical School. This study followed the Strengthening the Reporting of Observational Studies in Epidemiology (STROBE) reporting guideline.

This cross-sectional study analyzed changes in mass shootings during the COVID-19 pandemic by examining publicly available information about mass shootings in the US between January 1, 2014, and June 30, 2021, from the Gun Violence Archive, a repository of gun violence incidents collected from over 7500 law enforcement, media, government, and commercial sources.^5^ In the data, mass shootings are defined as shootings in which 4 or more people were killed or injured, not counting the perpetrator (eMethods in the Supplement).

We downloaded the data between April 14, 2021, to July 2, 2021, and analyzed the data from April 14, 2021, to July 3, 2021. We plotted 28-day moving means of the count of mass shootings in each year between 2014 and 2020, with partial data from January to June 2021. We focused on the period from mid-April 2020 onward and compared the data with the trends in the years prior to account for any seasonal variation in mass shootings. In addition, we conducted an event study analysis with 2738 daily observations that modeled the daily count of mass shootings as a function of a quadratic time-trend and adjustments for the month of the year (indicator variables for each month) and the day of the week (indicator variables for Monday through Sunday) and assumed a postpandemic period that began on April 16, 2020.

To examine whether postpandemic changes in mass shootings were concentrated in particular cities, we organized the 882 cities in the data into 3 groups according to the frequency of prepandemic mass shootings, with each group accounting for one-third of the prepandemic mass shootings (18 cities were high frequency, 103 were medium frequency, and 761 were low frequency). Similar event study models were estimated within each subgroup. We used a significance threshold of *P* < .05 using a 2-tailed test. Stata version 15.1 (StataCorp) was used for statistical analyses. Additional analyses are described in the Supplement.

## Results

The data included 2985 mass shootings during the study period, involving 3185 people killed and 12 547 injured. Overall, 1604 (54%) of the events involved 4 people killed or injured; 745 (25%) involved 5 people killed or injured; 288 (10%) involved 6 people killed or injured; 162 (5%) involved 7 people killed or injured; and 186 (6%) involved 8 or more people killed or injured. An increase in mass shootings was observed from May 2020 onward compared with the trends in the years prior (Figure). For example, 88 shootings occurred in July 2020, 42 shootings occurred in July 2019, and 45 shootings occurred in July 2018. In an event study analysis, we estimated that following April 16, 2020, there were a mean of 0.78 (95% CI, 0.52-1.04) additional daily mass shootings, 0.49 (95% CI, 0.07-0.92) additional people killed daily, and 3.40 (95% CI, 2.07-4.72) additional people injured daily in mass shootings.

**Figure.**
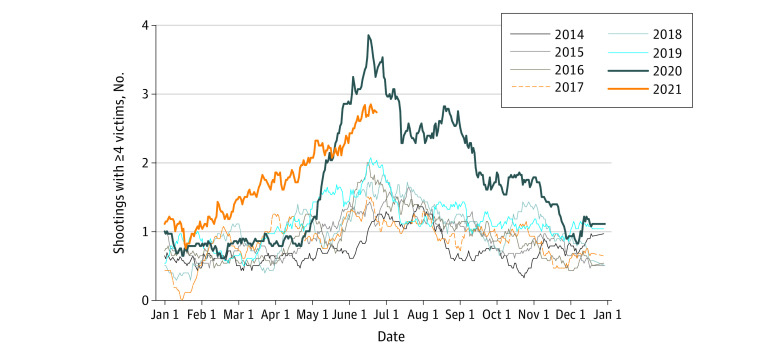
Daily Mass Shootings in the United States Twenty-eight-day moving mean. Mass shootings are defined as shootings in which 4 or more people were killed or injured, not counting the perpetrator. The moving mean includes less than 28 days but at least 14 days at the start and the end of the period covered.

Although increases in pandemic-related mass shootings were observed in each of the 3 groups of cities defined according to the prepandemic frequency of mass shootings, cities with low and high frequency of prepandemic mass shootings contributed disproportionately more to the overall increase in the number of people killed (Table). For example, the number of additional people killed daily was 0.26 (95% CI, 0.06 to 0.46) in high prepandemic frequency cities, −0.02 (95% CI, −0.27 to 0.23) in medium frequency cities, and 0.25 (95% CI, −0.03 to 0.53) in low frequency cities. Overall, the estimates suggest that over the 15 months analyzed there were 343 mass shootings above expected, involving an additional 217 people killed and 1498 people injured. Findings were robust to analyses that modeled alternative start dates (Table), alternative time trends (ie, linear, quadratic, cubic, and quartic), and additional definitions of mass shootings (ie, ≥5, ≥6, ≥7 people injured or killed).

**Table.** Daily Changes in Mass Shootings, People Killed, or People Injured Associated With COVID-19 Pandemic^a^

Group of cities according to prepandemic frequency of mass shootings	Modeled pandemic start date			
	March 16, 2020	April 1, 2020	April 16, 2020	May 1, 2020
**Change in No. of daily mass shootings (95% CI)**				
All	0.65 (0.39 to 0.91)	0.72 (0.46 to 0.98)	0.78 (0.52 to 1.04)	0.86 (0.60 to 1.12)
Low	0.23 (0.09 to 0.37)	0.27 (0.13 to 0.42)	0.29 (0.14 to 0.43)	0.32 (0.17 to 0.46)
Medium	0.23 (0.09 to 0.36)	0.25 (0.12 to 0.38)	0.27 (0.13 to 0.40)	0.31 (0.17 to 0.44)
High	0.20 (0.07 to 0.33)	0.20 (0.07 to 0.33)	0.22 (0.09 to 0.36)	0.24 (0.11 to 0.38)
**Change in No. of daily people killed (95% CI)**				
All	0.26 (−0.17 to 0.70)	0.39 (−0.04 to 0.82)	0.49 (0.07 to 0.92)	0.55 (0.12 to 0.98)
Low	0.12 (−0.17 to 0.40)	0.19 (−0.09 to 0.48)	0.25 (−0.03 to 0.53)	0.29 (0.01 to 0.58)
Medium	−0.05 (−0.31 to 0.21)	−0.04 (−0.29 to 0.22)	−0.02 (−0.27 to 0.23)	−0.03 (−0.28 to 0.23)
High	0.20 (−0.00 to 0.41)	0.23 (0.03 to 0.43)	0.26 (0.06 to 0.46)	0.28 (0.08 to 0.48)
**Change in No. of daily people injured (95% CI)**				
All	2.86 (1.52 to 4.20)	3.16 (1.83 to 4.50)	3.40 (2.07 to 4.72)	3.80 (2.48 to 5.13)
Low	0.92 (0.28 to 1.57)	1.09 (0.44 to 1.74)	1.14 (0.50 to 1.79)	1.28 (0.63 to 1.93)
Medium	1.25 (0.40 to 2.09)	1.39 (0.55 to 2.23)	1.45 (0.63 to 2.28)	1.65 (0.83 to 2.47)
High	0.69 (0.03 to 1.35)	0.68 (0.03 to 1.34)	0.8 (0.15 to 1.45)	0.89 (0.22 to 1.53)

^a^ Baseline results use April 16, 2020, as the starting date of the COVID-19 pandemic. The official statewide stay-at-home orders were issued at different dates between March 19, 2020, and April 7, 2020. For instance, California and New York issued such orders early (March 19 and 20, 2020), whereas Texas and Florida issued them relatively late (April 2, 2020). Additionally, the first peak in daily deaths in the US was reached on April 15, 2020. All regressions had 2738 observations (the number of days from January 1, 2014, to June 30, 2021) and included month fixed effects and day-of-week fixed effects. The 95% CIs were calculated using robust standard errors. Mass shootings were defined as shootings where 4 or more people were killed or injured, not counting the perpetrator.

## Discussion

This study found large increases in mass shootings in the US with the start of the COVID-19 pandemic consistent with the notion that mass shootings, an extreme form of violence, may be influenced by social and economic factors. However, this study was limited because the data analyzed were from a large gun violence data repository rather than a national database and focused on a specific form of gun violence.

## Notes

Supplement.
eMethods.
