# Supplementary material for: Mass Shootings in the US During the COVID-19 Pandemic
Source: JAMA Netw Open. 2021 Sep 16;4(9):e2125388. doi: 10.1001/jamanetworkopen.2021.25388 (PMC8446816; doi:10.1001/jamanetworkopen.2021.25388)
Supplement: Supplement. — eMethods. [file jamanetwopen-e2125388-s001.pdf]

Supplemental Online Content

Peña PA, Jena A. Mass shootings in the US during the COVID-19 pandemic. *JAMA Netw Open*. 2021;4(9):e2125388. doi:10.1001/jamanetworkopen.2021.25388

eMethods.

This supplemental material has been provided by the authors to give readers additional information about their work.

© 2021 Peña PA et al. *JAMA Network Open.*

eMethods.

We estimated the following multivariable linear regression model:

$$y_t = \theta_m + \eta_d + \beta x_t + \gamma t + \delta t^2 + \varepsilon_t \qquad (1)$$

Where y_t is the number of mass shootings that occurred on date t, θ_m is a month-of-year fixed effect that accounts for within-year fluctuations in the number of events that could be associated with seasonal events like weather, school breaks, etc. The fixed effects were estimated with separate indicator variables for each month (January, February, March, etc.). η_d is a day-of-week fixed effect that accounts for within-week fluctuations in the number of events that could be associated with different patterns across days (e.g., weekdays vs weekends). The fixed effects were estimated with separate indicator variables for each day of the week (Sunday, Monday, Tuesday, etc.). x_t is an indicator variable that is equal to zero for dates before April 16, 2020, and equal to one for dates on or after April 16, 2020. β is the parameter of interest and is interpreted as the average increase in daily mass shootings associated with the pandemic. The term $\gamma t + \delta t^2$ represents a quadratic trend in the number of mass shootings over the entire period of analysis, from January 2014 to June 2021. Such trend could be increasing, decreasing or flat, depending on the data. Lastly, ε_t is the error term.

Analogous models to (1) were used for the other two outcomes analyzed: number of people killed, and number of people injured. We used a significance threshold of .05 using a 2-sided test. Stata version 15.1 (StataCorp) was used for statistical analyses.

To create the database that we analyzed, Gun Violence Archive utilizes "automated queries, manual research through over 7,500 sources from local and state police, media, data aggregates, government and other sources daily." The database is publicly available and was downloaded on July 2, 2021. Although we have no way to verify the completeness of the Gun Violence Archive database, we have no empirical grounds to suspect it may have a longitudinal bias systematically related to the time elapsed since the start of the COVID-19 pandemic. For instance, the number of mass shootings reported in January 2021 is comparable to the number of shootings reported in January of previous years. This suggests the absence of any mechanical increase in reports of shootings after the COVID-19 pandemic started.

The 882 cities in the Gun Violence Archive data were organized into three groups, according to the frequency of pre-pandemic mass shootings (prior to March 2020). Each group accounted for one third of pre-pandemic mass shootings: 18 cities were high frequency (e.g., Chicago, IL, and St Louis. MO), 103 were medium (e.g., Austin, TX, and Cincinnati, OH), and 761 were low (e.g., Mesa, AZ, and Lexington, KY).

© 2021 Peña PA et al. *JAMA Network Open.*
